# Manual of Surgical Treatment
*By Sir W. Watson Cheyne, Bart., C.B., F.R.S., and F. F. Burghard, M.S., F.R.C.S. New edition, with the assistance of T. P. Legg, M.S., F.R.C.S., and Arthur Edmunds, M.S., F.R.C.S. Vol. II. (Longmans, Green & Co. 1912. Pp. 570. Price 21s.)


**Published:** 1912-06-08

**Authors:** 


### Manual of Surgical Treatment.*

The second volume of this magnificent text-book has appeared with commendable promptitude hard upon the heels of the first one. It deals with the surgical affections of the skin and subcutaneous tissues, nails, lymphatic glands and vessels, muscles, tendons and tendon sheaths, nerves, veins, arteries, and bones, concluding with a section upon amputations. Probably the changes which have been found necessary in editing the first edition have been less extensive in this volume than in the others; though in such matters as the operative treatment of fractures there is, of course, much that has changed. With respect to this latter question the authors preserve an excellently balanced judgment, neither underestimating the value of accurate apposition by screws and plates, nor exaggerating the difficulty of securing satisfactory results by conservative methods. Their general outline of principles in the treatment of fractures is a healthily sane and practical exposition of the *via media*, which will probably be the accepted outcome of present-day controversies. They are also in favour, it may be noted, of the immediate reduction of all fractures provided that the selected apparatus for retaining the fragments in apposition is at hand. Some authors prefer the plan of waiting twenty-four hours or so; although there is something to be said for this delay, the considerations which are in this volume advanced against it have prevailed with a great many surgeons in favour of earlier treatment. Another point which is well emphasised by the authors is the desirability of so manipulating the fractured ends of a bone that the jagged spikelets shall interlock in a position of good coaptation. This, in effect, secures a modified degree of impaction which is most valuable in the maintenance of a proper position. Due stress is also laid upon the use of *x*-rays, not merely for diagnostic purposes, but also to govern the setting of the fracture during that process. The plate representing the musculo-spiral nerve embedded in callus following fracture of the shaft of the humerus is a good one; but it seems supererogatory to print it in three separate places in the volume. Once would surely have been enough.

As in the first volume (and the first edition), the practical note is everywhere struck. No detail is omitted; and details often make all the difference between comfort and distress for the patient. Thus it may be asked, How often are the chest and axilla shaved in hospital before Sayre's strapping is applied for fracture of the collar-bone? And this is only one of numerous similar hints which are scattered freely through the book. The authors might, we think, have spoken rather more authoritatively about lymphangioplasty—an operation which has, we believe, disappointed the expectations even of its originator, Mr. Handley. As it is they give an impression of hesitation whether to praise or disparage it.

The decline of the formal amputation set in long ago; and it has proceeded rapidly during the last decade. Not only so, but amputations are themselves becoming quite rare outside classes and examinations in operative surgery. The same can be said of operations for aneurism; and it is, therefore, not surprising to see these two subjects, once the backbone of operative surgery, discussed at less comparative length than they would then have been. The favourable impression which we formed of the first volume of the new series is sustained and strengthened by the second; it is an epitome of current surgical practice which no operator should omit to study.

* By Sir W. Watson Cheyne, Bart., C.B., F.R.S., and F. F. Burghard, M.S., F.R.C.S. New edition, with the assistance of T. P. Legg, M.S., F.R.C.S., and Arthur Edmunds, M.S., F.R.C.S. Vol. II. (Longmans, Green & Co. 1912. Pp. 570. Price 21s.)

